# Factors related to married or cohabiting women’s decision to use modern contraceptive methods in Mahikeng, South Africa

**DOI:** 10.4102/phcfm.v10i1.1431

**Published:** 2018-10-11

**Authors:** Godswill N. Osuafor, Sonto M. Maputle, Natal Ayiga

**Affiliations:** 1 School of Health Science, University of Venda, South Africa; 2 Population and Health Research Focus Area, North-West University, South Africa

## Abstract

**Background:**

Sexual and reproductive decision-making has emerged as an important health indicator in family reproductive health issues. While there is evidence of male dominance in sexual and reproductive health decisions, the role of socio-demographic factors on women’s decision to use contraception is not well understood.

**Aim:**

This study aimed at exploring the socio-demographic factors associated with married women’s decision-making to use contraception.

**Setting:**

The study was conducted in Mahikeng local municipality in the Modiri Molema District Municipality.

**Methods:**

Data were generated in Mahikeng from married and cohabiting women, aged 18–49 years, from a survey comprising 568 participants. Data were collected on women’s demographic characteristics and contraceptive behaviour. Descriptive, bivariate and multivariate analyses were used to examine factors related to decision-making on contraceptive use.

**Results:**

The result revealed that 57% of the participants were currently using contraception and 45% stated jointly-made decision regarding the use of contraception. Decisions on use of contraceptives were associated with education, occupation, religion, duration of union and home language. Other factors associated with decision-making on contraceptive use were perception on husband’s right to sex, use of force for sex and spousal communication about sex.

**Conclusion:**

Empowering women to use contraception to meet their fertility desire should aim at improving their socio-economic status and spousal communication. Family planning providers should recognise socio-cultural barriers under which the relationships exist and how women can navigate these contextual factors.

## Introduction

The extent to which women participate in decision-making on family planning may have a positive influence in meeting their reproductive health goals. Women’s primacy in fertility and contraceptive use has been reported in previous research on fertility regulation.^1,2,3,4^ Women with a higher decision-making power and autonomy have few children^5,6,7^ and are more likely to meet their reproductive health goals.^6,8,9,10^ Previous studies^8,11^ have shown that women play a major role in decision-making regarding the use of contraception. Other studies reported that joint decision-making to use contraception was higher as compared to women who made individual decisions.^12,13^

Studies have shown that women’s participation in decision-making on family planning was associated with socio-economic status.^14,15,16,17,18^ These studies concluded that women who were economically disadvantaged and have limited formal education partake less in family planning decision-making and consequently may not reach their reproductive goals. Although education and economic advantage empower women, social or gender norms wield greater influence in reproductive health decision-making power.^10,19,20^ For instance, economic advantage did not enhance the reproductive decision-making power of women in Nigeria and Pakistan.^20,21^ In northern Nigeria, women were denied access to family planning services in government facilities because they did not provide consent evidence from their husbands.^22^ Furthermore, Grady et al.^19^ failed to support the notion that decision-making on contraception comes within the domain of women but rather depends on the type of union.

Several studies in South Africa and elsewhere have reported male dominance in reproductive decision-making,^8,21,22,23,24^ which partly accounts for unwanted pregnancies.^25,26^ The implication of men dominating reproductive decision-making is that women exercise their reproductive right covertly^25,26^ and hence do not have control over their reproductive lives. However, the covert practice of contraception certainly contradicts the reproductive health right and free choice to family planning.

Women’s participation in decision-making on contraceptive usage remains crucial in the control of their reproductive life amidst the cultural relevance and socio-economic values on fertility. Reproductive health rights emphasise women’s individual decision on when to have children and how many they want but fall short in accounting for the realities in decision-making on contraception, especially in marital relationships where cultural and socio-economic factors play critical roles. Socio-cultural groups are the main classifications in South Africa. Differences in cultural practices have implications on women’s reproductive decision-making. There is an improved access to family planning and women have an opportunity to work outside their homes. However, evidence of unwanted pregnancies, while the government invests heavily on women’s autonomy in contraceptive use, calls for investigating the factors related to decision-making on the use of modern contraceptive methods among married or cohabiting women in Mahikeng, South Africa. This study aimed at examining the pattern in modern contraceptive use and factors related to decision-making to use contraception in Mahikeng.

## Methods

### Study design

A cross-sectional descriptive study using a quantitative approach was conducted. The quantitative data provided a general understanding regarding decision-making on modern contraceptive use in Mahikeng municipality, North-West province of South Africa.

### Setting

The study area was the Mahikeng local municipality in the Modiri Molema district Municipality. Mahikeng, apart from being a local municipality, is also the capital of the North-West province and occupies an area of about 6465 km^2^. It comprises 31 wards within designated residential areas (urban) and 108 villages (rural). About 75% of Mahikeng local municipality is rural. The southern and western parts of the municipality constitute rural areas which are the tribal territories. Mahikeng and Mmabatho constitute the urban areas, which are the suburbs and 16 residential units. Mahikeng includes Danville, Imperial Reserve, Riviera Park, Golf View, Libertas, Rooigrond and the central business district, while Mmabatho consists of Leopard Park, Montshiwa, Extension 39, Extension 38 and the 16 units.

### Study population, sample size and sampling strategy

The enumeration demarcations of census 2011 were used as the primary sampling unit, which were treated as clusters (Statistics South Africa 2012). Each cluster had at least 150 households. Using simple random sampling, four clusters from rural and two from urban areas were selected. A total of 800 households were selected using a multi-stage sampling from the designated clusters. The unit of analysis within the household was married or cohabiting women of age 18–49 years. In the cases where there were more than one eligible respondent, one was randomly selected by balloting. Women who were not in a heterosexual union were excluded. The sample size for the study was determined using Yamane’s (1973) formula: *n* = *N/1+N(e)*,^2^ where *n* is the sample size, *N* is the population size and *e* is the level of precision. The sample size was calculated based on the extrapolated sampling frame of 41 278.5 currently married or cohabiting women in Mahikeng municipality: *n* = (41 278.5 )/(1+41 278.5 (0.05))^2, which yielded 397. To compensate for non-reachable and non-responses, 40% of the sample size was added, which produced a total sample size of 556. A total of 568 eligible respondents participated in the survey. The detailed sampling of the methodology has been published in an article called ‘Risky sexual behaviours among married and cohabiting women and its implication for sexually transmitted infections in Mahikeng’.^27^

### Data collection

The structured questionnaire was developed after a review of related studies. The questionnaire covered the demographic and reproductive health decision-making characteristics of the respondents. The main outcome variable was decision-making to use contraception. Women were asked the following question: ‘who decides when to use contraceptive method?’ The responses were categorised as a woman alone, (1) or jointly (2) and husband (0), where she did not partake in any decision-making. Several pre-cautionary measures were taken at every stage of data collection and entry. Research assistants were trained in line with the objective of the study. A small-scale trial of the study was carried out to pretest the accuracy of the questionnaire in answering the objectives. The interview was carried out face-to-face by eight research assistants who were trained in demographic and health survey data collection. During the data collection process, field editing was done by the researcher. The data were entered in an Excel spreadsheet. The accuracy of data entry was assessed by re-entering 250 questionnaires, randomly selected, from the 568 surveyed questionnaires. The cross-domain analysis was used to compare the data values in two columns to identify inconsistencies.

### Data analysis

Data processing was carried out by a statistician using the IBM Statistical Package for Service Solutions (SPSS) version 20. The data were imported from Excel sheets to SPSS and subsequently analysed at three levels. The first level was a description of the demographic profiles of the study respondents. The second level was a chi-square test of associations between decision-making to use contraception and socio-demographic characteristics of the respondents. The third level was a multinomial logistic regression model informed by the nature of the outcome variables. The results were presented as odds ratios and confidence intervals at 95% level of significance.

## Results

### Demographic characteristics of the respondents

Table 1 presents the socio-demographic characteristics of the 568 respondents. Over three-quarter of the respondents were living in the rural area and about 50% of the women were below the age of 35. Two-thirds of the respondents were speaking Setswana. Slightly above one-third were in a civil marriage. Slightly above 50% had primary or no education. Over a quarter of the respondents were unemployed. Over one-third belonged to the Pentecostal faith. About two-fifths of the respondents commenced their marriage or union at an age below 25 years and two-thirds have been in marriage or union for less than 10 years. Slightly above 50% had one or two living children. Over half of the respondents reported that their husbands or partners do not use force for sex. About two-thirds stated they have no difficulties regarding spousal communication about sex with their husbands.

**TABLE 1:** Percentage distribution of women by socio-demographic characteristics.

Characteristics	*N*	Percentage
**Residence**		
Rural	445	78.3
Urban	123	21.6
**Age group**		
< 25	64	11.3
25–29	107	18.8
30–34	114	20.1
35–39	120	21.1
40–44	90	15.8
45–49	74	13.0
**Home language**		
Setswana	376	66.2
Afrikaans	21	3.7
IsiXhosa	50	8.8
Sesotho	74	13.0
IsiZulu	47	8.3
**Type of union**		
Civil	212	37.3
Religion	112	19.7
Traditional	149	26.2
Cohabiting	95	16.7
**Highest educational level**		
No education	112	19.7
Primary	179	31.5
Secondary	156	27.5
Tertiary	121	21.3
**Occupation**		
Unemployed	169	29.8
Business and/or trading	76	13.4
Government worker	193	34.0
Student	47	8.3
Domestic worker and/or security	83	14.6
**Religion affiliation**		
Roman Catholic church	72	12.7
Methodist	146	25.7
Pentecostal	213	37.5
Seventh Day Adventist	95	16.7
Traditional religion	42	7.4
**Number of living children**		
None	77	13.6
1–2	290	51.1
3–4	168	29.6
5+	33	5.8
**Duration of the union**		
< 5	218	38.4
5–9	158	27.8
10+	192	33.8

Note: *N* = 568.

### Knowledge and current use of contraception by method type

**FIGURE 1:**
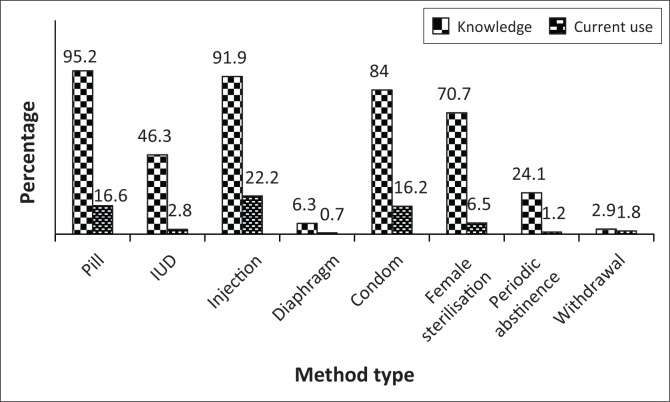
Distribution of knowledge and current use of contraception by method type.

Figure 1 presents the knowledge and current use of contraception by method types. Almost all respondents (99.9%) had knowledge about modern contraceptive methods and about 57.2% were currently using contraceptives. Over 90% had knowledge of pills and injections. Other known methods were condom use and female sterilisation. The most common use by method types were injections, pills and condoms. There is a big gap between the knowledge and current contraceptive usage among women.

### Relationship of decision-making to use contraception with socio-demographic characteristics of respondents

Forty-five per cent of the respondents reported that the decision to use contraception was jointly-made by the couple, whereas individual decision-making to use contraception was reported by 22% of the respondents. Table 2 shows the patterns in decision-making to use contraception by background characteristics. Apart from the age and use of force to have sex, chi-square analyses revealed an association between the patterns in decision-making and all other socio-demographic characteristics. Individual decision-making to use contraception was widespread for union duration of 10 years or more, whereas combined decision-making was most common if duration of union was less than five years. The proportion of women reporting individual or combined decision-making for the use of contraception was higher among Setswana and Afrikaans speaking women compared to husbands as decision makers. Women with primary education showed the highest percentage in stating their husbands as the decision-makers on contraceptive use, whereas those with no formal education had the lowest percentage in reporting individual decision-making on contraception. Highest percentages in reporting individual or combined decision-making on contraceptive use were observed among government workers. Women’s dominance in decision-making on contraceptive use was highest among women who professed Pentecostalism and reported ‘no’ to a husband’s right to sex. Combined decision-making on contraceptive use was common among women who engaged in spousal communication about sex with their husbands. The influence of the husband in decision-making on contraceptive use was higher among women who did not engage in spousal communication about sex.

**TABLE 2:** Percentage distribution of respondents by pattern of decision-making to use contraception and demographic characteristics.

Characteristics	Total (*N* = 568)	Husband and/or partner (*n* = 187)	Wife (*n* = 124)	Both (*n* = 257)	*p*
**Age group**	**-**	**-**	**-**	**-**	0.235
< 25	11.3	8.6	10.5	13.6	-
25–29	18.8	19.8	18.5	18.3	-
30–34	20.1	20.3	21.0	19.5	-
35–39	21.0	16.6	22.6	23.3	-
40–44	15.8	16.0	17.7	14.8	-
45–49	13.0	18.7	9.7	10.5	-
**Duration of union**	**-**	**-**	**-**	**-**	0.035
< 5	38.4	39.6	33.1	40.1	-
5–9	27.8	20.9	30.6	31.5	-
10+	33.8	39.6	36.3	28.4	-
**Home language**	**-**	**-**	**-**	**-**	0.022
Setswana	66.2	59.4	70.2	69.3	-
Afrikaans	3.7	2.7	2.4	5.1	-
IsiXhosa	8.8	11.8	7.3	7.4	-
Sesotho	13.0	13.4	10.5	14.0	-
IsiZulu	8.3	12.8	9.7	4.3	-
**Educational level**	**-**	**-**	**-**	**-**	< 0.000
No education	19.7	25.7	16.9	16.7	-
Primary	31.5	40.6	29.8	25.7	-
Secondary	27.5	17.1	29.8	33.9	-
Tertiary	21.3	16.6	23.4	23.7	-
**Occupation**	**-**	**-**	**-**	**-**	< 0.001
Unemployed	29.8	40.6	30.6	21.4	-
Business	13.4	12.8	9.7	15.6	-
Government worker	34.0	27.3	35.5	38.1	-
Students	8.3	3.7	11.3	10.1	-
Domestic worker and/or security	14.6	15.5	12.9	14.8	-
**Religion**	**-**	**-**	**-**	**-**	< 0.000
Roman Catholic church	12.7	7.5	21.8	12.1	-
Methodist	25.7	28.3	19.4	26.8	-
Pentecostal	37.5	37.4	37.9	37.4	-
Seventh Day Adventist	16.7	13.4	18.5	18.3	-
Traditional religion	7.4	13.4	2.4	5.4	-
**Partner has right to use force for sex**	**-**	**-**	**-**	**-**	< 0.000
No	76.6	64.2	83.1	82.5	-
Yes	8.3	14.4	6.5	4.7	-
I do not know	15.1	21.4	10.5	12.8	-
**Partner uses force for sex sometimes**	**-**	**-**	**-**	**-**	0.804
No	51.2	51.9	53.2	49.8	-
Yes	48.8	48.1	46.8	50.2	-
**Spousal discussion about sex**	**-**	**-**	**-**	**-**	< 0.000
Difficult	35.7	50.3	36.3	24.9	-
Not difficult	64.3	49.7	63.7	75.1	-

### Multivariate analysis of the factors related to women’s decision-making on the use of contraception

Table 3 presents multivariate analyses conducted to identify factors related to women’s participation in contraceptive use decision-making. Women who had secondary education were more likely than those without formal education to state that they make decisions alone on contraceptive use, compared to the husband being the decision-maker. Students compared to unemployed women were more likely to report that they make a decision to use contraception. Marital duration below 5 years compared to 5–9 years showed 60% reduced ability in stating female contraceptive use, decision-making relative to the husband making the decision. Those that belong to the Methodist, Pentecostal, Seventh Day Adventist (SDA) and other religions were less likely to report who take part in decision-making to use contraceptions, compared to Roman Catholic women. Women who reported ‘I do not know’, compared to those who said ‘no’ as to whether partner had a right to sex, showed 53% reduced ability in making an individual decision on contraceptive use, relative to the husband as a decision-maker.

**TABLE 3:** Multinomial regression showing the factors related to decision-making to use contraception.

Variables	Women		Both	
	Odds ratio	95% CI	Odds ratio	95% CI
**Education**				
No education (ref)	1000	-	-	-
Primary	1.106	0.541–2.263	0.748	0.413–1.353
Secondary	2.368*	1.016–5.517	1.807	0.901–3.624
Higher and/or tertiary	2.043	0.640–6.525	1.494	0.573–3.895
**Occupation**				
Unemployed (ref)	1000	-	1000	-
Business and/or trading	1.027	0.433–2.439	2.400*	1.203–4.788
Government worker	1.650	0.847–3.214	2.198**	1.239–3.897
Student	4.110*	1.382–12.225	3.740**	1.400–9.991
Domestic worker	0.801	0.367–1.747	1.337	0.698–2.561
**Religion**				
Roman Catholic Church (ref)	1000	-	1000	-
Methodist	0.185**	0.079–0.433	0.458*	0.211–0.990
Pentecostal	0.362*	0.147–0.894	0.717	0.309–1.662
Seventh Day Adventist	0.331**	0.152–0.725	0.560	0.267–1.178
Traditional religion	0.073**	0.017–0.307	0.292*	0.107–0.792
**Duration of union**				
< 5	0.403**	0.209–0.777	0.537*	0.309–0.931
5–9 (ref)	1000	-	1000	-
10+	0.689	0.368–1.288	0.469**	0.270–0.814
**Home language**				
Setswana (ref)	1000	-	1000	-
Afrikaans	0.995	0.199–4.989	1.908	0.565–6.438
IsiXhosa	0.469	0.193–1.139	0.550	0.268–1.130
Sesotho	0.777	0.359–1.680	1.117	0.602–2.073
IsiZulu	0.891	0.376–2.111	0.305**	0.130–0.717
**Partner has right to use force for sex**				
No (ref)	-	-	1000	-
Yes	0.432	0.171–1.090	0.399*	0.178–0.894
I do not know	0.470*	0.222–0.996	0.642	0.354–1.162
**Spousal discussion about sex**				
Difficult (ref)	1000	-	1000	-
Not difficult	1.373	0.741–2.541	2.399**	1.415–4.069
**Partner uses force for sex sometimes**				
No	-	-	1000	-
Yes	1.500	0.870–2.587	2.256**	1.414–3.597

Note: Data in bold indicates *p*-value significance.

ref, reference category; CI, confidence interval.

* , Significant at *p* < 0.05;

** , significant at *p* < 0.001.

Combined decision-making relative to the husband’s dominance was higher if the woman was a student, a government worker or had her own business, compared to being unemployed. Reporting shared decision-making relative to the husband’s dominance was lower (70%) if the women speak Zulu, compared to Setswana-speaking women. In comparison to the women who were in marital union for a period of 5–9 years, women with a marital duration of less than 5 years and 10 years or more had, respectively, 53% and 46% reduced odds of participating in combined decision-making to use contraception, relative to husbands as decision-makers. Compared to women who stated the absence of spousal communication about sex, odds of combined decision-making to use contraception were higher among those who reported practising spousal communication. Combined decisions relative to husband as decision-maker to use contraception among those who professed Methodism and other religions were lower, compared to Roman Catholic women. Reporting combined decision-making rather than the husband making the decision on contraceptive use was observed among women who stated that partners use force for sex, compared to women who were not forced. Women who reported that husbands have a right to sex relative to those who disagreed with this statement, showed 60% reduced odds in combined decision-making compared to husbands making decisions for contraceptive.

## Discussion

This study revealed that almost all the women had knowledge of modern contraceptive methods. However, the magnitude of the usage was not commensurate with the knowledge. The figure on current use was lower than the 60% for national contraceptive prevalence.^28^ This may be attributed to the differences in decision-making power to use modern contraception. Most of the decisions on the use of modern contraception were jointly made, which is consistent with studies in Kenya and Nigeria.^12,16^

Consistent with previous studies, women with secondary education were more likely to exercise autonomy in decision-making to use contraception.^14,29,30,31^ Formal education of women can spearhead decision-making through exposure to the importance of contraceptive use. Furthermore, it empowers women to use innovative ideas such as family planning through the power of knowledge. However, tertiary education was not a significant predictor of decision-making to use contraception. This may partly be attributed to the small number of women who had tertiary education. Women’s economic status also impacts their decision-making regarding contraceptive usage. The higher likelihood of employed women being able to make decisions to use contraception is consistent with other studies.^12,15^ Individual decision-making to use contraception by married women who were students can be explained within the desire to postpone childbearing to gain higher education. The economic status of women in the study empowered them to have a say in decision-making on contraceptive use.

In this study, the lower likelihood of women who professed Methodism, Pentecostalism, SDA and traditional religion may suggest religious and cultural inequality in decision-making between husband and wife, even when the woman is affected. Men are supposed to be heads of families and most religious women are expected to communicate their intentions to their husbands. Where there was disagreement, it is likely that the husband’s decision may prevail. Thus, religious belief has continued to be a hindrance in women’s ability to use contraception. Different religions have perceived contraceptive use as an act of contravening the injunction of God to pro-create and be fruitful. Furthermore, highly religious women do not accept unnatural birth control measures and thus the unwillingness to participate in contraceptive use decision-making.^32^ Other factors such as willingness to be pregnant, fear of side effects and opposition from the husbands could also affect contraceptive decision-making.

Women’s ability to decide to use contraception was associated with the duration of the union. It was not unexpected that shorter duration into marriage was associated with low female or combined decision-making on the use of contraceptives because it is still early to exercise such individual decision-making. It could be envisaged that a shorter duration of union coincides with the beginning of their reproductive life, which makes the need for contraception less necessary. Duration of the union over 10 years was associated with low combined decision-making on contraceptive use for women. Perhaps as the relationship advances, women invest excessively in the sustainability of the relationship by relinquishing their decision-making power. Thus, having a say in shared decision-making on contraceptive use for fertility control may not be an area of interest to them.

Participation of women in decision-making to use contraception may have a bearing on their cultural orientation. The lower chances of women who speak IsiZulu in participating in decision-making on contraceptive use may suggest a power imbalance between husband and wife attributed to cultural influences of male dominance in reproductive decision-making. The findings are consistent with previous studies^6,21^ that documented that culture may have an influence on women’s contraceptive use decision-making power. Women’s ability to participate in decision-making on contraceptive use was compromised by acceptance and ignorance as to whether husbands have a right to sex. This can be explained on the ground of cultural socialisation which advances men to be superior to their wives. Women acknowledging that the husbands have a right to sex may represent submissiveness of women to the oath of marital sexual tenets. This may or will have some implication in women reproductive health.

The finding that positive spousal communication was associated with enhancing women’s decision-making on contraceptive use is in agreement with other studies.^13,33,34^ It is anticipated that through spousal communication about sexual matter, the need to regulate fertility is communicated to their partners. Other studies have indicated that the use of contraception was high among women who discussed contraceptive matters with their partners.^13,33^ Thus, this study emphasises the need for information, education and communication (IEC) between couples for reproductive health.

It was unexpected that being forced to have sex was found to be associated with joint decision-making on contraception. The use of force for sex in steady relationships may affect the self-esteem of women and compromise control over their reproductive lives. However, the higher ability of women who were coerced to have sex may support a previous study in Eastern Cape, South Africa, where women who reported physical violence by partners showed higher contraceptive use than other women.^31^ Thus, forced to have sex may be a bargaining ground for the women to strike a balance in decision-making on contraception.

### Limitations

The findings of the study are pertinent for women’s health promotion in terms of contraceptive use. However, there are some caveats in interpreting the findings. Data collected were based on self-reporting, which pose some difficulties in response verification. A strategy to minimise this shortcoming was by asking questions for the opinions of respondents rather than their personal experiences. Another limitation is that the study was conducted among predominantly Setswana-speaking people. Hence, small samples of other ethnic groups cannot substantively reflect cultural ideology of the people on contraceptive use decision-making. These limitations were considered when interpreting the findings. However, survey research on structural and socio-cultural influences on women’s contraceptive use decision-making has produced reliable and consistent results which agree with our findings.

### Recommendation

Programmes aimed at empowering women to decide freely on when and what type of contraception to use should take into consideration the ethnic diversity of South Africa. There is a need to involve religious leaders in any programme aimed at promoting women’s contraceptive use adoption. Given that shared decision-making is most ideal in marital or cohabiting relationships, sexual health programmes should focus on improving spousal communication as partners influence the contraceptive decisions of women.

## Conclusion

South Africa is undergoing socio-cultural changes in which the level of women’s participation in sexual and reproductive decision-making has prominence. We conclude that traditional attitudes to sex and reproductive issues continue to revolve on a belief that men had the upper hand in sexual matters. The short duration of union and religion promote poor women’s participation in decision-making to use contraception. On the other hand, occupation and spousal communication about sex enhanced women’s participation in contraceptive use decision-making.
