# Corrigendum: Factors related to married or cohabiting women’s decision to use modern contraceptive methods in Mahikeng, South Africa

**DOI:** 10.4102/phcfm.v10i1.1998

**Published:** 2018-12-11

**Authors:** Godswill N. Osuafor, Sonto M. Maputle, Natal Ayiga

**Affiliations:** 1 School of Health Science, University of Venda, South Africa; 2 Population and Health Research Focus Area, North-West University, South Africa; 3 Department of Social Work, Kabale University, Uganda

In the version of this article published earlier, the third author’s affiliation was misstated. The affiliation of Natal Ayiga is hereby updated and corrected to the ‘Department of Social Work, Kabale University, Uganda’. The author sincerely regrets this error and apologises for any inconvenience caused.

